# Deep Learning for Alzheimer’s Disease Prediction: A Comprehensive Review

**DOI:** 10.3390/diagnostics14121281

**Published:** 2024-06-17

**Authors:** Isra Malik, Ahmed Iqbal, Yeong Hyeon Gu, Mugahed A. Al-antari

**Affiliations:** 1 Department of Computer Science, COMSATS University Islamabad, Wah Campus, Wah Cantt 44000, Pakistan; 2 Department of Computer Science, Sir Syed Case Institute of Technology, Islamabad 45230, Pakistan; 3 Department of Artificial Intelligence and Data Science, College of AI Convergence, Daeyang AI Center, Sejong University, Seoul 05006, Republic of Korea

**Keywords:** Alzheimer’s disease, brain diseases, dementia, computer-aided diagnosis (CAD) system, machine learning, deep learning

## Abstract

Alzheimer’s disease (AD) is a neurological disorder that significantly impairs cognitive function, leading to memory loss and eventually death. AD progresses through three stages: early stage, mild cognitive impairment (MCI) (middle stage), and dementia. Early diagnosis of Alzheimer’s disease is crucial and can improve survival rates among patients. Traditional methods for diagnosing AD through regular checkups and manual examinations are challenging. Advances in computer-aided diagnosis systems (CADs) have led to the development of various artificial intelligence and deep learning-based methods for rapid AD detection. This survey aims to explore the different modalities, feature extraction methods, datasets, machine learning techniques, and validation methods used in AD detection. We reviewed 116 relevant papers from repositories including Elsevier (45), IEEE (25), Springer (19), Wiley (6), PLOS One (5), MDPI (3), World Scientific (3), Frontiers (3), PeerJ (2), Hindawi (2), IO Press (1), and other multiple sources (2). The review is presented in tables for ease of reference, allowing readers to quickly grasp the key findings of each study. Additionally, this review addresses the challenges in the current literature and emphasizes the importance of interpretability and explainability in understanding deep learning model predictions. The primary goal is to assess existing techniques for AD identification and highlight obstacles to guide future research.

## 1. Introduction

**Figure 1:**
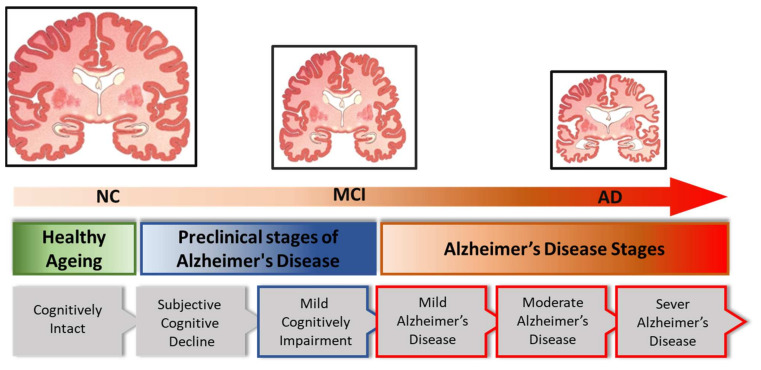
Alzheimer brain structure differences with the progress of brain diseases: normal control, mild cognitive impairment (MCI) brain, and an Alzheimer’s disease (AD) brain.

There are various possible causes of Alzheimer’s disease, a progressive brain disorder that affects memory, thinking, and behavior of elder age males and females. The exact cause of Alzheimer’s is not fully understood, and it is likely that the disease is caused by a combination of factors, including genetics, environmental influences, and lifestyle [1]. Dementia is a general term that is generated from Latin, with ‘de’ indicating ‘apart’ and ‘mentis’ indicating ‘mind’. Dementia damages nerve cells, causing a decline in memory, confusion, a decline in thinking and language skills, behavioral changes, and changes in other mental abilities that eventually lead to death due to trauma [2]. Dementia is divided into different categories, like Alzheimer’s, Lewy bodies, cardiovascular, frontotemporal dementia, Parkinson’s disease dementia, and Wernicke–Korsakoff syndrome. Alzheimer’s disease directly affects some parts of the brain that allow humans to perform common body actions like hiking, swallowing, and eating. In many advanced states, it is one of the most costly diseases, and it places physical and psychological burdens on caregivers. In an early stage, the diagnosis of AD is necessary for proper treatment. Alzheimer’s disease (AD) is uncommon in people at age 47. Earlier diagnosis mostly depends on the assessment of the patient’s past time, medical report, or mental evaluation [3]. Currently, there are no nominal and competent diagnostic tools accessible for diagnosing AD. There is no experiment that can verify whether a person has AD or not; while surgeons can assess whether an individual has dementia or not, the actual reason can be hard to control. Dementia causes the brain to lose mass, and the difference in size is clearly depicted in Figure 1 as a comparison between a normal control (NC) brain, a mild cognitive impairment (MCI) brain, and an Alzheimer’s disease (AD) brain. In individuals with normal cognition, there is typically minimal to no significant brain shrinkage. Those with mild cognitive impairment (MCI) might experience brain volume reductions of about 1–2% per year, which is faster than normal aging. However, individuals with Alzheimer’s disease experience brain volume reductions of approximately 3–5% per year. Specific regions, such as the hippocampus, can shrink even faster, sometimes up to 10–15% per year in advanced stages.

According to the Alzheimer’s Association, Alzheimer’s disease is the sixth leading cause of death in the United States [4]. As of 2021, it is estimated that there are approximately 6 million Americans living with Alzheimer’s disease, and this number is expected to increase to almost 14 million by 2060 [5]. Additionally, it is estimated that one in three seniors die with Alzheimer’s disease or another form of dementia. These statistics highlight the importance of increasing awareness and funding for research into the prevention, treatment, and cure of Alzheimer’s disease. One of the main factors that is thought to contribute to the development of Alzheimer’s is age. The risk of developing Alzheimer’s increases with age, and the disease is most common in people over the age of 65 [6]. Other potential causes of Alzheimer’s include the following:

- Genetics: Certain genetic variations have been identified that may increase the risk of developing Alzheimer’s.
- Environmental factors: Exposure to certain toxins or head injuries may increase the risk of developing Alzheimer’s.
- Lifestyle factors: Poor nutrition, lack of physical activity, and other unhealthy lifestyle habits may increase the risk of developing Alzheimer’s.
- Medical conditions: Certain medical conditions, such as high blood pressure, diabetes, and high cholesterol, may increase the risk of developing Alzheimer’s.

Nowadays, deep learning has been successfully applied to various tasks in medical image analysis, such as image classification, object detection, and segmentation. Therefore, researchers are focusing more on DL models to improve the performance of medical applications and reduce the time and cost of the diagnostic process. Multiple reviews and surveys have been published on Alzheimer’s disease and diagnosis methods to help new researchers propose a novel method. Saleem et al. [7] reviewed the most recent datasets and biomarkers used for diagnosis of Alzheimer’s disease. Researchers have reported that deep learning techniques such as feed-forward DNN, CNN, AE, RNN, DBN, GAN, and hybrid DL are widely used for AD diagnosis. The imaging modalities MRI, fMRI, fNIRS, FDG-PET, amyloid-PET, Tau-PET, EEG, and MEG are important for the AD diagnosis process. Khojaste-Sarakhsi et al.’s [8] survey covers a comparative analysis of 90 published papers that present deep learning architectures. Another review [9] was conducted to review Alzheimer’s disease detection models built using federated learning. Federated learning allows hospitals to train these models together without sharing private patient data. The study analyzes existing models, including their design, data use, and challenges for future improvement. Researchers [10] developed a standardized set of filters for radiomics, a field that extracts features from medical images. This improves the reliability of radiomics tools used by doctors. They identified eight filter types and created reference images and feature values for verification. A web tool allows software developers to check their tools for compliance with the standards.

In this review, self-supervised learning and explainable model attention mechanisms are considered. This review provides recommendations for conducting new research targeting Alzheimer diagnosis. Taneveer et al. [11] reviewed 165 papers on Alzheimer’s disease published between 2005 and 2019. Support vector machines (SVMs), artificial neural networks (ANNs), deep learning (DL), and ensemble methods are the primary areas under which machine learning approaches are evaluated. Amir et al. [12] presented the most recent findings and trends after conducting a thorough literature evaluation of more than 100 articles. Regarding handling neuroimaging data arising from single-modality and multimodality investigations, they review useful biomarkers and features (personal information, genetic information, and brain scans), the necessary pre-processing stages, and various approaches. Similarly, Al-Shoukry et al.’s [13] mini-review paper covers Alzheimer datasets, diagnosis technique biomarkers, and neuroimaging techniques for image analysis. Motivated by previously published work, we present the latest review to cover brain imaging modalities, feature extraction techniques, datasets, proposed methods, tools, evaluation measures, and validation techniques. The remainder of the paper is organized as follows: Section 2 presents search strategies. Section 3 presents details on datasets widely used in Alzheimer’s disease detection. Section 4 covers SVM-based machine learning applications for Alzheimer’s disease. Section 5 presents deep learning approaches for AD detection. Section 6 and Section 7 present the discussion and the review paper’s outcomes.

## 2. Search Strategy

**Figure 2:**
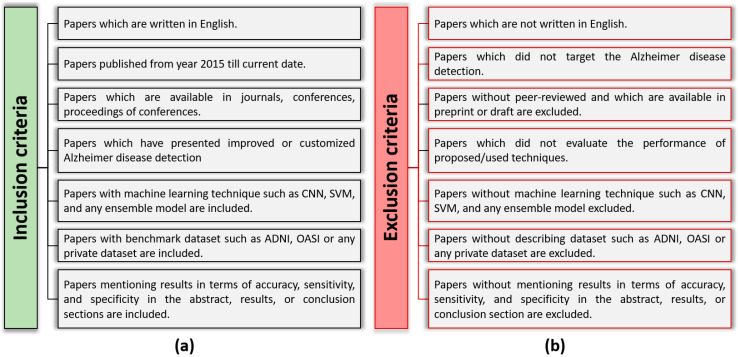
(**a**) shows the inclusion criteria, and (**b**) depicts the exclusion criteria of conducting this review.

We searched for important research papers using Google scholar, Scopus, Web of Science, PubMed, and ScienceDirect, which are all freely available services. Research papers that did not cover classification performance were excluded. Our search query was designed as follows: “(Alzheimer OR dementia) AND (disease OR sickness OR illness OR disorder) AND (detection OR classification OR detect) AND (technique OR method OR approach OR framework OR trends)”. We also designed specific inclusion/exclusion criteria for papers, as presented in Figure 2.

By using a combination of synonyms and related terms connected by OR, the query ensures a wide coverage of relevant papers, capturing different terminologies used by various researchers. The use of AND ensures that all aspects of the query must be present in the papers, thereby narrowing down to studies specifically discussing the detection and classification of Alzheimer’s or dementia. Including terms like “technique”, “method”, “approach”, “framework”, and “trends” helps in pinpointing papers that delve into the technical aspects of detection and classification, which are crucial for understanding the performance and efficacy of these methods. The search strategy ensures that a wide range of relevant literature is included, avoiding the exclusion of important studies due to varied terminology; focuses on the core areas of interest—detection and classification techniques—ensuring the gathered papers are pertinent to the review; and helps in quickly filtering out irrelevant papers that do not discuss classification performance, thus saving time during the review process.

The search query returned the following results: Google Scholar (22,200), Science Direct (33,838), Scopus (11,123), PubMed (3963), and Web of Science (1386). After the application of predefined inclusion/exclusion criteria, we retrieved 106 research papers from Elsevier (45), IEEE (25), Springer (19), Wiley (6), PLOS One (5), MDPI (3), World Scientific (3), Frontiers (3), PeerJ (2), World Scientific (3), Hindawi (2), IO press (1), and some other multiple sources (2).

## 3. Alzheimer Datasets

Several datasets are publicly available that are used by researchers to evaluate Alzheimer methods.

### 3.1. ADNI Dataset

In 2003, the “ADNI dataset” was established as a publicly available dataset on its website [14]. The ADNI dataset provides all information about patients having AD or not and mild cognitive impairment (MCI). It mainly considers observing various affected person information like the time of life, gender, and education. The ADNI dataset is used to detect AD at an early stage. The core purpose of the ADNI dataset is to test MRI(t) and PET biomarkers and to perform scientific and cognition psychophysiology evaluation in combination to assess the progression of mild MCI [13].

ADNI offers a wealth of information beyond just diagnoses. It includes MRI and PET scans, genetic data, cognitive test results, and cerebrospinal fluid (CSF) analysis. This multimodal approach allows researchers to look for a combination of factors that might indicate early AD. ADNI has been collecting data since 2004, with participants undergoing repeated assessments over time. This longitudinal aspect is crucial for capturing the gradual progression of Alzheimer’s and identifying subtle changes that might precede major symptoms. The richness of ADNI data can also be a challenge. Analyzing and integrating information from various sources requires sophisticated techniques and expertise. Alzheimer’s is presented differently in individuals. The ADNI dataset may not fully capture this variability, potentially limiting the generalizability of findings.

### 3.2. OASIS Dataset

The OASIS dataset is based on a group of 416 individuals ranging in age from 18 to 96 [15,16]. Three or four distinct T1-weighted MRI scans performed in a single scan session are presented for each subject. Men and women, both right-handed, are represented among the subjects. One hundred of the over-60 participants had received a clinical diagnosis of very mild to moderate Alzheimer’s disease (AD). A reliability dataset is also supplied, which contains 20 nondemented participants who were photographed 90 days after their initial session on a second visit. OASIS provides MRI scans and some clinical data at no cost, making it a good starting point for researchers, especially those with limited budgets. The OASIS dataset includes individuals across a spectrum of cognitive function, from healthy to those with Alzheimer’s. This allows researchers to study the progression of brain changes associated with early decline. OASIS primarily focuses on MRI scans, lacking the richness of data offered by ADNI (PET scans, genetic data, etc.). This can limit the ability to explore the multifaceted nature of Alzheimer’s. OASIS has fewer participants overall, and specifically fewer with early-stage Alzheimer’s. This can lead to issues with statistical power when detecting subtle early changes. The selection criteria for OASIS participants might not perfectly reflect the broader population, potentially introducing bias into the findings.

### 3.3. The Harvard Medical School Dataset

The HMS dataset includes T2-weighted brain MIR data [17]. The size of these images is 265 by 256 pixels. These 613 images are divided into two Alzheimer’s disease classes; 27 images belong to the normal class, and 513 to the abnormal. The normal class has two cases, while the abnormal class has forty cases. In the present HMS dataset, abnormal images are related to provocative diseases, neoplastic, degenerative, and cerebrovascular. HMS specifically targets individuals deemed non-cognitively impaired at baseline. This focus on the early stages of Alzheimer’s makes it directly relevant for early detection research. HMS includes data collected over multiple years, allowing researchers to track changes in brain function and structure as participants progress. This longitudinal aspect is crucial for capturing the early stages of Alzheimer’s disease. The HMS dataset is freely available, promoting collaboration and wider participation in early detection research. Compared to ADNI and OASIS, HMS is a newer project. This means there might be less data available at present, limiting the scope of analyses. While the HMS website mentions a publicly available dataset, details about specific data types and access procedures might be less readily available compared to established resources like ADNI and OASIS.

### 3.4. Max Planck Institute Leipzig Mind-Brain-Body Dataset

“This dataset is publicly available and includes 227 healthy participants comprising a young (N = 153, 25.1 ± 3.1 years, range 20–35 years, 45 female) and an elderly group (N = 74, 67.6 ± 4.7 years, range 59–77 years, 37 female) acquired cross-sectionally in Leipzig, Germany, between 2013 and 2015 to study mind–body–emotion interactions. During a two-day assessment, participants completed an MRI at 3 Tesla (resting-state fMRI, quantitative T1 (MP2RAGE), T2-weighted, FLAIR, SWI/QSM, DWI) and a 62-channel EEG experiment at rest. During task-free resting-state fMRI, cardiovascular measures (blood pressure, heart rate, pulse, respiration) were continuously acquired.

Techniques like image flipping and rotation can artificially expand datasets and reduce overfitting. Assigning higher weights to the under-represented class during training can balance the model. Pre-training models on a larger, more diverse dataset can improve performance on smaller datasets like ADNI or OASIS. Testing the model on completely independent datasets ensures generalizability and reduces bias. The specific methods for ensuring data reliability and validity can vary depending on the dataset (ADNI, OASIS, HMS) and the research itself. However, researchers generally employ several strategies to address these concerns. Researchers [13] verify the credibility of the data source itself. For established datasets like ADNI and OASIS, this might involve reviewing the institutions and protocols behind data collection. Techniques are used to identify and address errors, inconsistencies, or missing values within the data. Researchers [13] perform initial analyses to visualize the data and identify any outliers or unexpected patterns that might indicate data quality issues.

## 4. Feature Selection and Extraction with SVM

Feature-selection-based methods normally play an essential part in classifying data. Similarly, various features are merged to form a single vector in different studies [18,19]. In a study [20], an SVM-based classifier is utilized for the accurate classification of AD subjects using brain volume and clinical data. Subjects were randomly assigned to a training group (AD = 46, normal = 46) and a testing group (AD = 45, normal = 46) for SVM modeling and validation, respectively. The highest result was 62.64% accuracy using the hippocampus volume alone. Mendonça et al. [21] proposed a novel method using graph kernels constructed from texture features captured from sMR images. In this approach, FreeSurfer was first used to segment MR brain images into various regions. Then, three different methods were used to extract 22 texture features, and the probability distributions of those features were used to determine the graph-node properties. With the use of extracted sagittal plane slices from 3D MRI images, a study [22] presented a DL model for all-level feature extraction and fuzzy hyperplane-based least square twin support vector machine (FLS-TWSVM) for the classification of the derived features for early diagnosis of AD (FDN-ADNet). In another study [23], for better prediction of AD, an ensemble-based generic kernel is proposed where master–slave architecture is hybridized to achieve optimum performance. The proposed model is an ensemble of Extreme Gradient Boosting, Decision Tree, and SVM_Polynomial kernel (XGB + DT + SVM). In a study [24], the application of a fully automatic CAD system based on supervised learning techniques to segmented brain magnetic resonance imaging (MRI) from ADNI participants for automatic categorization was suggested. Two important qualities of the suggested CAD system are its optimal performance and visual aids for decision-making. In [25], a system for combining edge and node characteristics for AD classification using multiple kernels is presented. Using ten-fold cross-validation, an assessment of the proposed method was carried out using MRI scans of 710 participants (230 healthy control (HC), 280 MCI (including 120 MCIc and 160 MCInc), and 200 AD participants) from the Alzheimer’s disease neuroimaging project database. Long et al. [26] presented a machine learning approach to compute and analyze the regional morphological changes of the brain between groups in order to distinguish patients with AD or moderate cognitive impairment (MCI) from healthy old and to predict AD conversion in MCI patients. An embedding algorithm and a learning strategy for classification were used after a symmetric diffeomorphic registration to calculate the distance between each pair of subjects.

The research presented in [27,28,29] focuses on finding the most effective model for detecting biomarker genes associated with AD using several feature selection methods, including mRMR (Minimum Redundancy Maximum Relevance) and ReliefF. By comparing these methods with an SVM classifier, these studies assessed the efficiency of feature selection techniques like mRMR, CFS, the chi-square test, F-score, and GA, using a benchmark AD gene expression dataset of 696 samples and 200 genes.

We selected 53 studies based on SVM-based techniques; all technique, year, modality, feature extraction, dataset, method, tool, measure, and validation details are presented in Table 1.

## 5. Deep Learning Approach Applications

Artificial neural networks (ANNs) are largely adopted for machine learning models that can model highly nonlinear patterns of data. Deep neural networks (DNNs) are more complex neural networks with multiple convolution operations, batch normalization, ReLU, and SoftMax functions. In this review, we will also discuss transfer learning, feature extraction, and deep learning ensemble methods.

### 5.1. Transfer Learning

Samples from only a single domain are normally used in conventional machine learning models, but performance is affected badly when samples are very small. Transfer learning is a method that makes use of samples from many auxiliary (related) domains in addition to the target domain. To improve performance in differentiating MCI-C from MCI-NC, Cheng et al. [70] presented a unique strategy for concurrently utilizing data from the auxiliary domain (i.e., AD and NC) and unlabeled data. Li et al. [3] also transferred knowledge gained from ADNI samples to the samples acquired locally through the subspace alignment algorithm. Orouskhani et al. [71] use a unique deep triplet network as a metric learning strategy for Alzheimer’s disease detection and brain MRI analysis. Because there are not enough samples, the suggested deep triplet network adds a conditional loss function to increase the model’s precision. Chui et al. [72] proposed a generative adversarial network (GAN) to generate additional training data in the minority classes of the benchmark datasets. Kumar et al. [73] proposed a scheme for efficiently retrieving significant characteristics from MRI (magnetic resonance imaging) medical images to diagnose Alzheimer’s at the MCI level. They suggested a classification model that employs the AlexNet architecture. Shanmugam et al. [74] presented a study that employed neuroimages and transfer learning to identify early signs of AD and different phases of cognitive impairment (TL). In this classification, 6000 photos from the ADNI database were used to train and test three pre-trained networks, including GoogLeNet, AlexNet, and ResNet-18.

### 5.2. Feature Selection Techniques

Many techniques have been proposed for better feature selection (FS) from neuroimaging data. Wang et al. [75] adopted a hybrid PSO with the artificial bee colony (ABC) optimization algorithm along with a feed-forward neural network (FFNN). This technique is utilized to deal with the problem of high dimensionality in whole-brain analysis by extracting features from specific ROIs of the brain. Gorji et al. [76] proposed a novel and effective technique based on pseudo-Zernike moments (PZMs) for the structural MRI-based diagnosis of MCI in persons from AD and healthy control (HC) groups. To extract discriminative information from the MR images of the AD, MCI, and HC groups, the proposed technique employed PZMs. The data retrieved from the MRIs were classified using two different artificial neural network types, based on learning vector quantization (LVQ) networks and pattern recognition, respectively. Jha et al. [77] extract features from an image and present the dual-tree complex wavelet transform (DTCWT). Principal component analysis is used to reduce the dimensionality of the feature vector (PCA). To separate AD and HC from the input MR images, the feed-forward neural network (FNN) is given the reduced feature vector. Liu et al. [78] present a deep multitask multichannel learning scheme for AD classification using MRI data. In order to extract several image patches around discovered landmarks, a data-driven method was used to retrieve discriminative landmarks from MR images. Mahendran et al. [79] adopted a technique for categorizing AD patients; a deep learning-based classification model with an embedded feature selection strategy was applied. The data were preprocessed by performing quality control, normalization, and downstream analysis before choosing the pertinent features. EL-Geneedy et al. [80] proposed a pipeline based on deep learning for the accurate diagnosis and stage stratification of AD. The suggested analytic pipeline makes use of 2D T1-weighted MR brain images and shallow convolutional neural network (CNN) architecture. In addition to a quick and precise AD diagnostic module, the suggested pipeline offers both a global classification (normal vs. mild cognitive impairment (MCI) vs. AD) and a local classification. Lahmiri et al. [81] introduced a convolutional neural network (CNN) model to automatically extract deep traits from magnetic resonance images (MRIs) without the need for any prior assumptions. Filtering is also used to reduce the number of features, and the k nearest neighbors (kNN) algorithm is used to distinguish between AD subjects and healthy control (HC) subjects. The Bayesian optimization (BO) algorithm is used to optimize the kNN. In a study [82], a hybrid EEG-fNIRS model for categorizing four classes of participants, comprising two groups of AD patients and two groups of healthy controls (HCs), was proposed. A linear discriminant analysis (LDA) classifier was used to assess the performance of EEG-derived and fNIRS-derived features after they had been sorted using a Pearson correlation coefficient-based feature selection (PCCFS) technique. In [83], the hybrid EEG-fNIRS was used in developing machine learning (ML)-based classification models to categorize four subject groups, including healthy controls (HCs) and three AD patient classes. For the multiclass classification using the fNIRS and EEG characteristics, a conventional neural network and a hybrid CNN and LSTM networks were developed. To implement binary and ternary illness classification models, three-dimensional convolutional neural networks (3D-CNNs) [84] were combined with magnetic resonance imaging (MRI). In order to compare the deep learning performances of 3D-CNN, 3D-CNN support vector machine (SVM), and two-dimensional (2D) CNN models, the dataset from the Alzheimer’s disease neuroimaging initiative (ADNI) was employed. In [85], the use of deep neural networks, in particular CNNs combined with saliency maps, trained on power modulation spectrogram inputs to find optimal patches in a data-driven manner, was proposed. Experiments were performed on EEG data acquired from 54 participants, including 20 healthy controls, 19 patients with mild AD, and 15 moderate-to-severe AD patients. Alzheimer’s disease (AD) poses significant challenges in early diagnosis, particularly in the mild cognitive impairment stages. Combining MRI and PET imaging can enhance diagnostic accuracy by leveraging MRI’s structural insights and PET’s physiological data. This paper introduces a multimodal fusion approach using discrete wavelet transform (DWT) and a pre-trained VGG16 neural network to optimize image analysis, reconstructing fused images with inverse DWT and classifying them using a vision transformer. An evaluation of the approach on the ADNI dataset achieved notable accuracies: 81.25% for MRI and 93.75% for PET in distinguishing AD from early and late mild cognitive impairment stages. Another paper [86] introduces a multimodal fusion approach utilizing the discrete wavelet transform (DWT) to analyze neuroimaging data. The optimization of this method is enhanced through transfer learning with a pre-trained VGG16 neural network.

The study [87] employs a spectral graph attention model to aggregate node embeddings within and between clusters of normal and diseased populations. This is followed by a bilinear aggregation model, which highlights abnormalities across different population categories. Finally, an adaptive fusion module dynamically combines the results from both models to improve Alzheimer’s disease (AD) prediction accuracy. In [88], the authors propose a heterogeneous ensemble framework of Bayesian-optimized time-series deep learning models to identify progressive deterioration of brain damage. The work [89] introduces a novel end-to-end coupled-GAN (CGAN) architecture for Alzheimer’s disease (AD) diagnosis. The CGANC network comprises two components: a CGAN for extracting fused features from multimodal MRI and PET data and a CNN for classifying these features. The CGAN is trained to encode both MRI and PET images into a shared latent space, from which fused features are extracted and classified into specific AD stages.

We selected 43 studies based on deep learning-based techniques; all technique, year, modality, feature extraction, dataset, method, tool, measure, and validation details are presented in Table 2.

## 6. Ensemble-Based Learning Approach Applications

Ensemble-based learning approaches have been widely used in the field of Alzheimer’s disease (AD) research to improve the accuracy and reliability of diagnosis, prediction, and classification models. Ruiz et al. [114] propose a four-way classification of 3D MRI images using an ensemble implementation of 3D DenseNet models. In this research, dense connections were used that enhance the movement of data within the model due to having each layer connected with all the subsequent layers in a block. Pan et al. [115] proposed a classifier ensemble developed by combining CNN and EL, i.e., the CNN-EL approach, to identify subjects with MCI or AD using MRI. A sizable number of CNN models were trained using a set of sagittal, coronal, or transverse MRI slices for each binary classification task before being combined into a single ensemble. An et al. [116] presented an ensemble learning-based approach for Alzheimer’s disease classification. This research outlines a novel application of machine learning to improve Alzheimer’s disease primary care. Fang et al. [117] introduced an approach that combines three state-of-the-art deep convolutional neural networks (DCNNs) with multimodality images for AD classification. Furthermore, they suggested a novel ensemble DCNN-based Adaboost algorithm-based multimodality data fusion and classification approach. Using stacked convolutional neural networks (CNNs) and a bidirectional long short-term memory (BiLSTM) network, El-Sappagh et al. [118] present a robust ensemble deep learning model. The multimodal multitask model utilizes a fusion of five types of multimodal time-series data in addition to a set of background (BG) knowledge to jointly predict multiple variables. Hedayati et al.’s [119] research presents a method that consists of two main steps. Firstly, an ensemble of pre-trained autoencoder-based feature extraction modules is employed to generate image features from a 3D input image. Secondly, a convolutional neural network is utilized for diagnosing Alzheimer’s disease. Razzak et al. [120] proposed using an integrated deep ensemble learning framework to enhance the accuracy of predicting Alzheimer’s disease (AD) diagnosis. In contrast to DenseNet, the authors introduce a multiresolutional ensemble PartialNet that is specifically designed for AD detection utilizing brain MRIs. PartialNet integrates identity mappings, diversified depth, and deep supervision, which enables effective feature reuse and, consequently, improves learning. In [121], a new approach is proposed that combines ensemble learning with the MDR constructive induction algorithm to efficiently identify epistasis interactions related to Alzheimer’s disease (AD). Discovering such interactions is a major obstacle and has a significant impact on personalized medicine (PM). The ensemble learning techniques utilized in this framework include Random Forest (RF) with the Gini index and permutation importance, Extreme Gradient Boosting (XGBoost), and classification and regression trees (CARTs).

We selected 11 studies based on ensemble-based approaches; all technique, year, modality, feature extraction, dataset, method, tool, measure, and validation details are presented in Table 3.

## 7. Discussion

**Figure 3:**
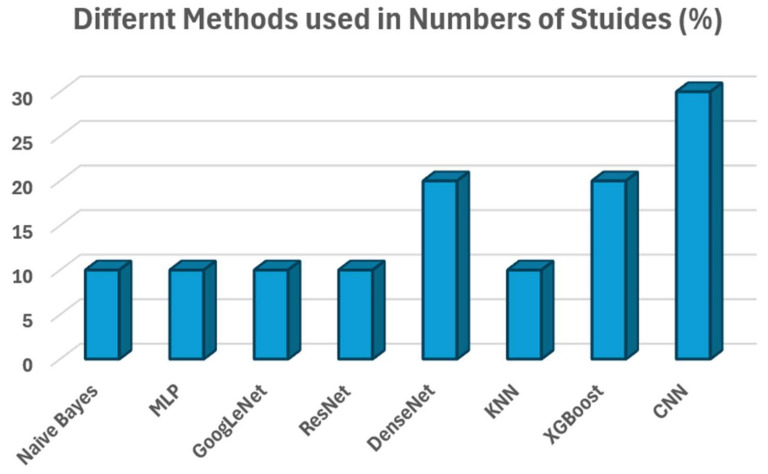
Plot displaying various methods used for ensemble-based learning approaches.

Our review paper results indicate that more than 90 studies used the most popular ADNI dataset. In second place, the OASIS dataset is used in 6 studies, followed by 23 studies that used in-house private datasets. In our review, 43% of the studies use the LOOCV validation method with 30% and 10% shares of 10-fold and 5-fold cross-validation methods. Furthermore, more than 80% of studies does not disclose their cross-validation method. As discussed in the above section, different SVM variants have been utilized for the early detection of Alzheimer’s disease. We observed that 83% of papers use a standard SVM classifier. This can also indicate the high popularity and effectiveness of the SVM classifier in AD prediction. Similarly, TWSM (3%) and LSTSVM (3%) are used, followed by CSVM (1%) usage. CNN architecture (AlexNet, ResNet, DenseNet, and VGG16) is utilized in more than 70% of studies. However, LSTM, PRNN, and GAN networks are also used in 20% of the studies. Furthermore, 10% of the studies used customized CNN architecture for accurate AD classification. As shown in Figure 3, ensemble-based learning approaches with SVM and CNN architecture are widely used, followed by, DenseNet, and XGBoost. The results and statistics show that ensemble-based learning approaches also mainly focused on SVM and CNN-based architectures.

**Figure 4:**
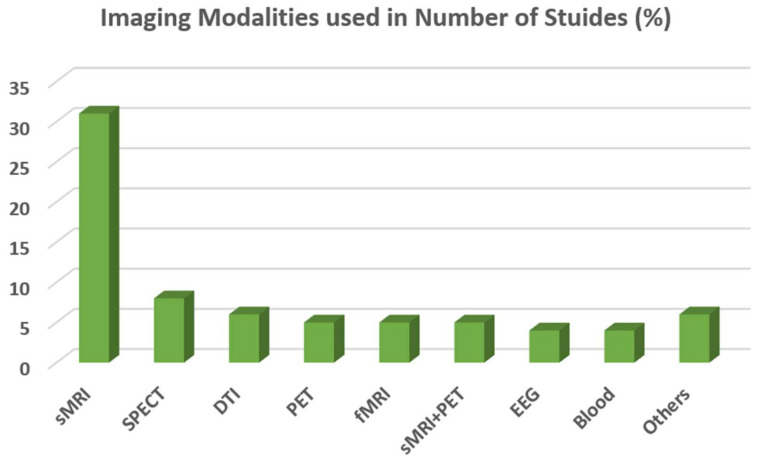
Graph illustrating different image modalities and other data utilized by SVM and deep learning techniques for Alzheimer’s.

In deep learning studies, we also noticed that in some studies, pre-trained models are used for the feature extraction process, and final classification is performed using SVM-based classifiers. Image modality plays a vital role in the classification of MRI-based images. T1-weighted images are used in the case of structural MRI (sMRI) images, and only a few studies use T2-based images [54]. This is because the delineation of the ventricular surface of the brain due to atrophy is clearly visible in T1-weighted images. Figure 4 displays the use of various modalities of data in the task of classifying Alzheimer’s using SVM. It can be seen that the sMRI modality is widely utilized, appearing in more than 30 research studies.

Transfer learning and data augmentation are suitable solutions for tackling over-fitting issues in DL models. We also identified that models developed on multimodal MRI (fMRI and DTI) perform superior to models developed on individual fMRI and DTI. Selecting the appropriate pre-processing and segmentation techniques is crucial for building efficient DL models for AD diagnosis. In this study, we noticed that the use of neurophysiological data with MRI and PET can improve an AD classification method. The hippocampus is an important ROI for AD diagnosis, and hippocampal atrophy is the most essential part for AD diagnosis. In summary, the choice between SVM, ensemble learning, and CNNs depends on the specific needs of the Alzheimer’s detection task. SVMs may be a good choice for simpler binary classification problems, while ensemble learning and CNNs may be better suited for more complex data or image analysis tasks. Ultimately, it is important to choose the algorithm that best fits the specific task and available resources. Overall, fNIRS has shown promising results in detecting early signs of Alzheimer’s disease and monitoring disease progression. However, further research is needed to validate the use of fNIRS in clinical settings and to develop robust and reliable algorithms for analyzing fNIRS data. Incorporating power analysis and functional network analysis into CAD systems holds immense potential for revolutionizing Alzheimer’s disease diagnosis and comprehension. Power analysis sheds light on minute, localized changes in brain activity, while functional network analysis reveals the broader impact of AD on brain connectivity. By working in tandem, these techniques pave the way for significantly more accurate and earlier detection of the disease, ultimately facilitating the development of personalized diagnostic and therapeutic strategies. This integration not only sharpens diagnostic precision but also offers a deeper dive into the neurological underpinnings of Alzheimer’s disease. Some papers might have employed methods to visualize the features the model focuses on when making detections. This could involve techniques like Grad-CAM (Gradient-weighted Class Activation Mapping), which highlights the image regions most influential in the model’s decision. Some studies might have conducted experiments where they remove or modify specific features within the model and observe the impact on detection accuracy. This helps understand which features are most critical for the model’s performance.

### Limitations and Future Work

Among the modalities commonly used in Alzheimer’s disease research, magnetic resonance imaging (MRI) is often considered the best-suited modality for this purpose. MRI provides high spatial resolution and excellent soft tissue contrast, allowing researchers to detect subtle structural changes in the brain associated with Alzheimer’s disease. Additionally, MRI can be used to evaluate multiple aspects of brain structure and function, such as white matter integrity, gray matter volume, and cortical thickness. In most studies, MRI and PET are the most used imaging modalities for Alzheimer’s disease diagnosis and monitoring. MRI is better suited for detecting structural changes, while PET is better suited for detecting molecular changes in the brain. However, the choice of imaging modality depends on the specific clinical question being addressed. The integration of these neuroimaging techniques can aid in identifying Alzheimer’s disease and can be combined with other factors such as memory test scores and genetic information to achieve a more precise diagnosis. Multiple multimodal fusion-based approaches have also contributed to improving the accuracy of classification. Fusion involves providing various inputs to a single network using different types of datasets, such as sMRI, PET, and fMRI, to obtain higher accuracy. While MRI and PET scans are the most used multimodalities for Alzheimer’s disease diagnosis, some studies have also incorporated neuropsychological test data and pathological data such as MMSE, CDR, and ADAS-Cog. These scores have been shown to increase classification accuracy by approximately 2%, as demonstrated in the preceding section. Although researchers have made significant strides in the early diagnosis of novel biomarkers, accurately predicting whether non-convertible mild cognitive impairment will become convertible mild cognitive impairment, and using multimodality for Alzheimer’s disease prognosis, there is still much work to be done. An approach can be developed for classifying multimodal data and clinical test data to further enhance classification accuracy. Including several neuropsychological tests and clinical data with other imaging data may also lead to improved classification and detection accuracy. Additionally, besides structural and functional neuroimaging modalities, biochemical functioning-based modalities such as magnetic resonance spectroscopy (MRS) can be integrated. MRS may aid in improving Alzheimer’s disease diagnosis by discovering new biomarkers that complement structural imaging modalities. This could enhance the prognosis capability for Alzheimer’s disease and increase the scope of differential dementia diagnosis.

Furthermore, the area of feature selection is also being researched for improvement. Choosing the area of interest instead of allocating the entire imaging data would undoubtedly enhance performance. The slice-based approach has been widely used, but it results in many feature arrays. As a result, the image can be segmented based on the area of interest, and the segmented data or extracted patches can be used to train the model. This approach would be more computationally efficient and less expensive than the entire slice-based learning. Additionally, we can include cerebral atrophies such as a decrease in GM and WM volumes, gyri shrinkage, sulcus expansion, and other structural deformations caused by AD, and treat each region as a separate area of interest to increase our model’s training ability. The Hyperparameter optimization approach can also be used to select learnable hyperparameter values of the network. The classification of MCI and CN subjects is the most challenging task, as accuracy is significantly lower than other classifications, as seen throughout the review. Measures can be taken to improve the classification from ncMCI to cMCI. This scope has attracted researchers’ attention, and much research is still ongoing in this domain. The window size selection and trial extraction can affect the performance of deep learning models for Alzheimer’s detection. Optimal window size and trial extraction methods depend on several factors, including the imaging modality, the research question, and the size of the dataset. Therefore, careful selection and optimization of these parameters are crucial for developing accurate and robust models for Alzheimer’s detection. The model over-fitting challenges are seen in both SVM and ANN models when the dataset has few samples and is more sensitive to noise. Another issue occurs, when the number of features for each data point exceeds the number of training samples, in this scenario SVM model underperform. Deep learning models also require extensive amount of label data, due to hospital ethical and privacy patient restriction policy make it difficult to access labeled data that can be major stumbling point in advancement of deep learning method for AD diagnosis. However, we also noticed that unsupervised deep learning techniques such as auto-encoders are effective for limited data challenges. The settings of hyperparameters like learning rate, drop-out, number of epochs, batch size, momentum, etc. have an impact on how well DL algorithms work. To obtain the same experimental outcome, it is imperative to apply the same set of hyper-parameters across a variety of levels. Developing explainable CNN models that can provide insights into the features and regions of the brain that are important for Alzheimer’s detection can improve clinical understanding and guide treatment decisions. Incorporating longitudinal imaging data into CNN models can improve the accuracy of disease prediction and help identify biomarkers for disease progression. Developing robust and secure systems for deploying CNN models in clinical settings is crucial for realizing their potential for improving patient outcomes. In future, conventional machine learning techniques (Random Forest, KNN, and SVM) can be utilized to assist DL network feature selection and discrimination processes.

Despite significant advancements, some limitations still exist concerning diagnosis and prognosis. Patients who suffer from claustrophobia or epilepsy are not suitable candidates for MRI procedures. Furthermore, researchers require additional multimodal data with continued follow-up to achieve more precise training, even after MRI scans are available. Ignoring neurodegeneration caused by age is a significant limitation because it is challenging to predict the extent of degeneration for each patient accurately. These limitations highlight the challenges surrounding the neuroimaging diagnosis of Alzheimer’s disease.

## 8. Conclusions

In this review, most studies employ three major machine learning methods—SVM, ANN, and ensemble-based learning approaches—for diagnosing Alzheimer’s disease. Researchers are also exploring advanced techniques such as transfer learning, ensemble learning, and multi-kernel strategies for SVM. Findings indicate that SVM is widely used due to its robustness. However, many studies note that ANN-based models often encounter the problem of local minima. Despite this, ANNs are highly adaptable for incremental learning, modeling sequential data, and quantizing high-dimensional spaces. Consequently, novel ANN variations may be beneficial for Alzheimer’s diagnosis, as deep learning and ensemble learning demonstrate promising results in accurately modeling highly complex data. Nonetheless, further research is needed to better integrate feature selection methods with machine learning models for specific data modalities. Additionally, we observed that most researchers focus more on feature extraction processes than on improving classification methods. Addressing this challenge in future studies could provide deeper insights into Alzheimer’s disease. Moreover, there is a need for developing machine learning models that can integrate data from multiple modalities for early detection of Alzheimer’s disease.

## Figures and Tables

**Table 1:** Comparison of cutting-edge systematic review research in terms of modality, feature extraction, datasets, methods, tools, evaluation metrics, and validation.

#	Reference	Year	Modality	Feature Extraction	Dataset	Method	Tools	Measures	Validation
**1.**	Khedher et al. [24],	2015	sMRI (T1)	VBM + fast ICA	ADNI	SVM (linear)	_	Acc = 77.62; Sens = 80.27; Spec = 74.49	2-fold
**2.**	Cabral et al. [30]	2015	FDG-PET	VI	ADNI	SVM, GNB	_	Acc = 80.0; Sens = 83.4; Spec = 76.4	10-fold
**3.**	Schmitter et al. [31]	2015	sMRI (T1)	VBM + VolBM	ADNI	SVM (linear)	_	Sens = 86; Spec = 91	LOOCV
**4.**	Zhang et al. [19]	2015	sMRI (T1)	VolBM + 3DDWT + PCA	_	SVM (RBF)	MATLAB	Acc = 81	5-fold
**5.**	Zhang et al. [32]	2015	sMRI (T1)	DF+PCA	OASIS	TWSVM	MATLAB	Acc = 92.75; Sens = 90.56; Spec = 93.37	10-fold
**6.**	Zhang et al. [33]	2015	sMRI (T1)	PCA	OASIS	SVM (polynomial)	MATLAB	Acc = 92.36	10-fold
**7.**	Xu et al. [34]	2015	sMRI	Lasso features	ADNI	S-LSTSVM(RBF)	MATLAB	Acc = 92.1; Sens = 92.52; Spec = 92.07	5-fold
**8.**	Retico et al. [35]	2015	sMRI (T1)	VBM + SVM-RFE	ADNI	SVM (linear)	-	Acc = 70.7;	20-fold
**9.**	Ortiz et al. [36]	2015	FDG-PET + sMRI	VBM + SICE	ADNI	SVM (linear)	-	Acc = 92; Sens = 96; Spec = 86	10-fold
**10.**	Zhu et al. [37]	2016	sMRI	PIS	ADNI	TS-SVM	_	Acc = 82.5	10-fold
**11.**	Khazaee et al. [38]	2016	rs-fMRI	Atlas (NBF)	ADNI	SVM	MATLAB	Acc = 87.29, 72.03, 97.46	Hold-out
**12.**	Suk et al. [39]	2016	sMRI (T1), PET, CSF	Atlas	ADNI	DW-S2MTL, SVM (linear)	_	Acc = 95; Sens = 92; Spec = 98	10-fold
**13.**	Plocharsky et al. [40]	2016	sMRI (T1)	Morpholgical features (length, area, depth)	ADNI	SVM (linear)	MATLAB	Acc = 87.9; Sens = 90; Spec = 86.7	10-fold
**14.**	Tong et al. [41]	2016	sMRI (T1) + FDG-PET + CSF + genetics	Atlas	ADNI	NGF + SVM	_	Acc = 91.8; Sens = 88.9; Spec = 94.7	Leave-p-ou
**15.**	Zhu et al. [42]	2016	sMRI (T1) + PET + CSF	Atlas	ADNI	SVM + LDA + LPP	_	Acc = 95.5	_
**16.**	Alam et al. [43]	2017	sMRI (T1)	VolBM + KPCA	ADNI	SVM (multiple kernels)	_	Acc = 93.85; Sens = 92.1; Spec = 94.45	10-fold
**17.**	Liu et al. [25],	2017	sMRI (T1)	Atlas	ADNI	MKBoost + SVM	_	Acc = 94.65; Sens = 95.03; Spec = 91.76	10-fold
**18.**	Khedher et al. [24]	2017	sMRI (T1)	ICA	ADNI	SVM(RBF)	_	Acc = 87.12; Sens = 89.92; Spec = 83.98	k-fold
**19.**	Beheshti et al. [44]	2017	sMRI (T1)	VBM+GA	ADNI	SVM (linear)	_	Acc = 93.01; Sens = 89.13; Spec = 96.8	10-fold
**20.**	Long et al. [26]	2017	sMRI (T1)	MDS+PCA	ADNI	SVM (linear)	MATLAB	Acc = 97.1; Sens = 93.85; Spec = 87.37	10-fold
**21.**	Tangaro et al. [45]	2017	sMRI (T1)	VolBM	ADNI	SVM (linear)	_	Acc = 100	10-fold
**22.**	Asgari et al. [46]	2017	Word count	LIWC	_	SVM+RF	_	Acc = 88.52; Sens = 84.60; Spec = 92.20	5-fold
**23.**	Hojjati et al. [18]	2017	rs-fMRI	PCC+F-score	ADNI	SVM (linear)	MATLAB	Acc = 91.4; Sens = 83.24; Spe = 90.1	9-fold
**24.**	Beheshti et al. [47]	2017	sMRI (T1)	VBM (NBF)	ADNI	SVM	_	Acc = 84.17; Sens = 88.83; Spec = 79.00	10-fold
**25.**	Alam et al. [48]	2017	sMRI	DTCWT/LDA	ADNI	TWSVM	MATLAB	Acc = 93.85; Sens = 92.1; Spec = 94.45	10-fold
**26.**	Sun et al. [49]	2018	sMRI (T1)	VBM+PCC	ADNI	Group lasso SVM	MATLAB	Acc = 95.1; Sens = 93.8; Spec = 83.8	5-fold
**27.**	Liu et al. [50]	2018	sMRI (T1)	Atlas	ADNI	MKBoost + SVM	_	Acc = 95.37; Sens = 92.49; Spec = 96.08	10-fold
**28.**	Zeng et al. [51]	2018	sMRI (T1)	PCA + PSO and SDPSO	ADNI	SVM (RBF)	_	Acc = 82.5	10-fold
**29.**	Basaia et al. [52]	2018	sMRI (T1)	GM, WM, CSF	ADNI	CNN	Python	Acc = 98.2; Sens = 98.1; Spec = 98.3	10-fold
**30.**	Lahmiri et al. [53]	2018	sMRI (T1)	VolBM	ADNI	SVM (polynomial)	_	Acc = 97.08; Sens = 98.09; Spec = 96.07	10-fold
**31.**	Kamathe et al. [54]	2018	sMRI (T1, T2) + PD	ICA	ADNI	SVM (polynomial)	MATLAB	Acc = 100	_
**32.**	Bi et al. [55]	2018	rs-fMRI	PCC	ADNI	RSVM (RBF)	_	Acc = 94.44	_
**33.**	Mazaheri et al. [56]	2018	EEG	TFRs	_	SVM (RBF)	_	Sens = 80; Spec = 95	LOOCV
**34.**	Paraskevaidi et al. [57]	2018	Blood plasma	PCA-LDA	_	SVM	MATLAB	Sens = 84; Spec = 86	LOOCV
**35.**	Hett et al. [58]	2018	sMRI (T1)	Hippocampal segments	ADNI	Fusion SVM	MATLAB	Sens = 93.4; Spec = 87.6	10-fold
**36.**	Peng et al. [59]	2019	sMRI + PET + SNP	Volume + mean intensity features		SVM (multiple kernels)		Acc = 96.1; Sens = 97.3; Spec = 94.9	10-fold
**37.**	Fritsch et al. [60]	2019	Linguistic data	n-gram		LSTM		Acc = 85.6	LOOCV
**38.**	Gosztolya et al. [61]	2019	Acoustic signal	MFCC	_	SVM (linear)	_	Acc = 80, Spec = 85.7	5-fold
**39.**	Xin Bi et al. [62]	2019	FMRI	ROI	ADNI	SVM, ELM, CNN	MATLAB	Acc = 94.44	5-fold
**40.**	Irie et al. [63]	2020	fNIRs	_	_	DL, NN	Python	Acc = 90	_
**41.**	Dachena et al. [64]	2020	FMRI, MMSE	ROI	ADNI	SVM	MATLAB	Acc = 95.65; Sens = 93.39; Spec = 97.22	_
**42.**	Burgos et al. [65]	2020	MRI, PET	ROI	ADNI	SVM, CNN	_	Acc	5-fold
**43.**	Sharma et al. [22]	2021	MRI, PET	ROI	ADNI	SVM	_	Acc = 72, 71, 48, and 91	LOOCV
**44.**	Vichianin et al. [20]	2021	MRI	ROI	ADNI	SVM	_	Acc = 62.64	_
**45.**	Mendonça et al. [21]	2022	MRI	ROI	ADNI	SVM	_	Acc = 92	5-fold
**46.**	Khan et al. [23]	2022	MRI	ROI	ADNI	SVM	_	Acc = 89.77	10-fold
**47.**	Sharma et al. [66]	2022	fNIRs	-	Private	SVM	_	Acc = 7.15, 97.29 and 95	LOOCV
**48.**	Arco et al. [67]	2023	fNIRs	-	Private	SVM	_	Acc = 98.95	LOOCV
**49.**	Pasnoori et al. [27]	2024	MRI	-	Kaggle	SVM, MRMR	_	Acc = 83.2	10-fold
**50.**	Demirhan et al. [28]	2024	MRI	-	Private	SVM, MRMR, ReliefF	_	Acc = 95.1	10-fold
**51.**	Alshamlan et al. [29]	2024	MRI	-	Private	SVM, MRMR, ReliefF	_	Acc = 83	10-fold
**52.**	Puri et al. [68]	2024	EEG	-	EEG dataset	SVM	_	Acc = 98.5	10-fold
**53.**	Pirrone et al. [69]	2024	EEG		EEG dataset	SVM, KNN	_	Acc = 95.5	5-fold

**Table 2:** Comparison of cutting-edge systematic review research in terms of modality, feature extraction, datasets, methods, tools, evaluation metrics, and validation.

#	Reference	Year	Modality	Feature Extraction	Dataset	Method	Tools	Evaluation	Validation
**54.**	Wang et al. [75]	2015	sMRI (T2)	DWT	-	ANN	MATLAB	Acc = 100; Sens = 100; Spec = 100	5-fold
**55.**	Cheng et al. [70]	2015	sMRI + (TC1S)F + PET	Atlas	ADNI	M2TL	_	Acc = 80.1; Sens = 85.3; Spec = 73.3	0-Fold
**56.**	Payan et al. [90]	2015	MRI	3D patch	ADNI	SAE	_	Acc = 95.38, 86.84, 92.11	_
**57.**	Gorji et al. [76]	2015	sMRI (T1)	Pseudo-Zernike moment	ADNI	PRNN, LVQNN	_	Acc = 97.27; Sens = 96.64, Spec = 97.79	10-fold
**58.**	Hosseini-Aslet al. [91]	2016	3D sMRI	VBM	ADNI	CNN	_	Acc = 99.30; Sens = 100; Spec = 98.60	10-fold
**59.**	Suk et al. [92]	2016	rs-fMRI	ROI	ADNI	CNN	_	Acc = 89; Sens = 73; Spec = 60	LOOCV
**60.**	Ortiz et al. [93]	2016	sMRI (T1)	VBM	_	DBN	Python	Acc = 90; Sens = 86; Spec = 94	10-fold
**61.**	Aljovic et al. [94]	2016	Biomarkers	_	_	ANN	MATLAB	Sens, Spec	_
**62.**	Zheng et al. [95]	2016	MRI, PET	93 ROI		MMSDPN		Acc = 97.27; Sens = 97.32; Spec = 98.33	10-fold
**63.**	Sarraf et al. [96]	2017	rs-fMRI	Slice-based	ADNI	DL-CNN	Python	Acc = 100	5-fold
**64.**	Hon et al. [97]	2017	sMRI (T1)	_	OASIS	CNN(TL)	MATLAB	Acc = 96.25	5-fold
**65.**	Suk et al. [98]	2017	sMRI	93 ROI	ADNI	JLLR DeepESRNe	_	Acc = 91.02; Sens = 92.72; Spec = 89.94	10-fold
**66.**	Jha et al. [77]	2017	sMRI (T1)	VBM	OASIS	PCA + FFNN	MATLAB	Acc = 90.06; Sens = 92.0; Spec = 87.78	10-fold
**67.**	Leracitano et al. [99]	2017	EEG	PSD, epoch	ADNI	CNN	MATLAB	Acc = 95.5; 79.7; 73.35; 61.06	_
**68.**	Liu et al. [78]	2018	sMRI (T1)	Patch-based	ADNI	Multitask multichannel deep neural network	MATLAB	Acc = 93.7, Sens = 94.6, Spec = 93.2	5-fold
**69.**	Lu et al. [100]	2018	FDG-PET	ROI	ADNI	MDNN	_	Acc = 93.58; Sens = 91.54; Spec = 95.06	10-fold
**70.**	Li et al. [101]	2018	sMRI (T1)	Patch based	ADNI	DenseNet	MATLAB	Acc = 89.5; Sens = 87.9; Spec = 90.8	10-fold
**71.**	Cui et al. [102]	2018	sMRI (T1)	VBM	ADNI	ANN + BGRU	_	Acc = 89.69; Sens = 86.87; Spec = 92.58	5-fold
**72.**	Spasov et al. [103]	2019	sMRI (T1)	ROI, APOe4	ADNI	CNN	Python	Acc = 100; Sens = 100; Spec = 100	10-fold
**73.**	Wang et al. [104]	2019	sMRI (T1)	Volume	ADNI	3D-CNN	_	Acc = 93.61, 98.42, 98.83, 97.52	10-fold
**74.**	Chitradevi et al. [105]	2019	MRI	GM, WM, ROI	_	DL, CNN	_	Acc = 98; Sens = 95; Spec = 94	_
**75.**	Dua et al. [106]	2020	MRI	CDR, ASF, nWBV	OASIS	SVM, CNN, RNN, LSTM	ipython, TensorFlow	Acc = 89.75, 92.22	_
**76.**	Lella et al. [107]	2020	MRI		ADNI	ANN, SVM, RBF	_	Acc = 85	10-fold
**77.**	Mehmood et al. [108]	2020	MRI, PET	DKPCA	ADNI	SVM, SCNN	_	Acc = 99.05	6-fold
**78.**	Liu et al. [109]	2020	MRI	FSL, SPM	ADNI	SVM, CNN	_	Acc = 79	_
**79.**	Xia et al. [110]	2020	sMRI	ROI	ADNI	3D CNN, 3D CLSTM, RNN	_	Acc = 94.19	10-fold
**80.**	Orouskhani et al. [71]	2022	sMRI	Volume	OASIS	VGG16	_	Acc = 99.41	_
**81.**	Chui et al. [72]	2022	sMRI	Volume	OASIS	GAN	_	Acc = 94, 93, 95	5-fold
**82.**	Helaly et al. [111]	2022	sMRI	ROI	ADNI	GAN	_	Acc = 94.34	6-fold
**83.**	Kumar et al. [73]	2022	sMRI	ROI	OASIS	AlexNet	_	Acc	_
**84.**	Mahendran et al. [79]	2022	sMRI	ROI	ADNI	DRNN	_	Acc	5-fold
**85.**	Shanmugam et al. [74]	2022	sMRI	ROI	ADNI	GoogLeNet, AlexNet, and ResNet-18	_	Acc = 96.39, 94.08, and 97.51	5-fold
**86.**	EL-Geneedy et al. [80]	2023	sMRI	ROI	ADNI, OASIS	CNN	_	Acc = 99.68	5-fold
**87.**	Cicalese et al. [82]	2023	fNIRs	-	Private	CNN	_	Acc = 79.81	5-fold
**88.**	Ho et al. [83]	2023	fNIRs	-	Private	CNN	_	Acc = 71.01	-
**89.**	Feng et al. [84]	2023	sMRI	-	ADNI	CNN	_	Acc = 82.57; Acc = 89.76; Acc = 95.74	-
**90.**	Lopes et al. [85]	2023	fNIRs	-	Private	CNN	_	Acc = 72.81	5-fold
**91.**	Lahmiri et al. [81]	2023	sMRI	sMRI	ADNI	CNN	_	Acc = 94.96; Sens = 92.05; Spec = 96.62	5-fold
**92.**	Zhang et al. [112]	2023	fNIRs	-	Private	CNN	Python, Tensorflow, Keras	Acc = 70.83%, 76.92%, 80.77%	5-fold
**93.**	Jiao et al. [113]	2023	fNIRs	-	Private	CNN	Python	Acc = 70%,	5-fold
**94.**	Odusami et al. [86]	2023	sMRI	ROI	ADNI	VGG16	Tensorflow	Acc = 93.97	5-fold
**95.**	Yang et al. [87]	2023	sMRI	ROI	ADNI	GNN	Pytorch	Acc = 95.00	5-fold
**96.**	Rahim et al. [88]	2024	sMRI	ROI	ADNI	GRU-LSTM	Pytorch	Acc = 88.00	5-fold
**97.**	Choudhury et al. [89]	2024	sMRI	ROI	ADNI	CGAN	Tensorflow	Acc = 94.00	5-fold

**Table 3:** Comparison of state-of-the-art systematic review research regarding modality, feature extraction, datasets, methods, tools, evaluation metrics, and validation.

#	Reference	Year	Modality	Feature Extraction	Dataset	P. Method	Tools	P. Evaluation	Validation
** 95 **	Ruiz et al. [114]	2020	sMRI	ROI	ADNI	Ensemble	-	Acc = 83.33	10-fold
** 96 **	Pan et al. [115]	2020	sMRI	ROI	ADNI	Ensemble (SVM, SENet, CNN)	-	Acc = 84.00	5-fold
** 97 **	An et al. [116]	2020	sMRI	ROI	NACC UDS	Ensemble (Bayes Nets, Hoeffding Tree, J48, Random Forest, Naive Bayes, MLP)	-	Acc = 78.5	5-fold
** 98 **	Fang et al. [117]	2020	sMRI	ROI	ADNI	Ensemble (GoogLeNet, ResNet, DenseNet)	MATLAB	Acc = 98.72	-
** 99 **	Baglet et al. [122]	2020	sMRI	ROI	OASIS	LR, SVM, DT, and RF	Tensorflow	Acc = 86	5-fold
** 100 **	El-Sappagh et al. [118]	2020	sMRI	ROI	ADNI	CNN, BiLSTM	-	Acc = 92.62	-
** 101 **	Hedayati et al. [119]	2021	sMRI	ROI	ADNI	Ensemble CNN	-	Acc = 95, Acc = 90, Acc = 92.5	10-fold
** 102 **	Khoei et al. [123]	2021	sMRI	ROI	ADNI	Stacking-based ensemble	-	Acc = 96.5	5-fold
** 103 **	Razzak et al. [120]	2022	sMRI	ROI	ADNI	Ensemble (DenseNet, PartialNet)	-	Acc = 100.0, Acc = 99.26, Acc = 88.71, Acc = 98.23	5-fold
** 104 **	Chatterjee et al. [124]	2022	sMRI	ROI	OASIS	Voting ensemble	-	Acc = 96.4	5-fold
** 105 **	Shaffi et al. [125]	2023	fNIRs	-	Private	Ensemble (KNN, XGBoost, SVM)	-	Acc = 94.92	5-fold
** 106 **	Hamid et al. [121]	2023	fNIRs	-	Private	Ensemble (XGBoost, CART)	-	Acc = 0.6515–0.6649	10-fold

## Abbreviations

AD:	Alzheimer’s disease
AI:	Artificial intelligence
SVM:	Support vector machine
ANN:	Artificial neural network
DL:	Deep learning
CAD:	Computer-aided diagnosis system
ACC:	Accuracy
SEN:	Sensitivity
SPE:	Specificity
MRI:	Magnetic resonance imaging
ROI:	Region of interest

## References

[1] Ahmed S., Choi K.Y., Lee J.J., Kim B.C., Kwon G.-R., Lee K.H., Jung H.Y. (2019). Ensembles of Patch-Based Classifiers for Diagnosis of Alzheimer Diseases. IEEE Access.

[2] Liu F., Zhou L., Shen C., Yin J. (2014). Multiple Kernel Learning in the Primal for Multimodal Alzheimer’s Disease Classification. IEEE J. Biomed. Health Inform..

[3] Li W., Zhao Y., Chen X., Xiao Y., Qin Y. (2019). Detecting Alzheimer’s Disease on Small Dataset: A Knowledge Transfer Perspective. IEEE J. Biomed. Health Inform..

[4] Alzheimer’s Association (2016). 2016 Alzheimer’s Disease Facts and Figures. Alzheimer’s Dement..

[5] Matthews K.A., Xu W., Gaglioti A.H., Holt J.B., Croft J.B., Mack D., McGuire L.C. (2019). Racial and Ethnic Estimates of Alzheimer’s Disease and Related Dementias in the United States (2015–2060) in Adults Aged ≥65 Years. Alzheimer’s Dement..

[6] Alzheimer’s Association (2010). 2010 Alzheimer’s Disease Facts and Figures. Alzheimer’s Dement..

[7] Saleem T.J., Zahra S.R., Wu F., Alwakeel A., Alwakeel M., Jeribi F., Hijji M. (2022). Deep Learning-Based Diagnosis of Alzheimer’s Disease. J. Pers. Med..

[8] Khojaste-Sarakhsi M., Haghighi S.S., Ghomi S.M.T.F., Marchiori E. (2022). Deep Learning for Alzheimer’s Disease Diagnosis: A Survey. Artif. Intell. Med..

[9] Sharma S., Guleria K. (2023). A Comprehensive Review on Federated Learning Based Models for Healthcare Applications. Artif. Intell. Med..

[10] Whybra P., Zwanenburg A., Andrearczyk V., Schaer R., Apte A.P., Ayotte A., Baheti B., Bakas S., Bettinelli A., Boellaard R. (2024). The Image Biomarker Standardization Initiative: Standardized Convolutional Filters for Reproducible Radiomics and Enhanced Clinical Insights. Radiology.

[11] Tanveer M., Richhariya B., Khan R.U., Rashid A.H., Khanna P., Prasad M., Lin C.T. (2020). Machine Learning Techniques for the Diagnosis of Alzheimer’s Disease. ACM Trans. Multimed. Comput. Commun. Appl..

[12] Ebrahimighahnavieh M.A., Luo S., Chiong R. (2020). Deep Learning to Detect Alzheimer’s Disease from Neuroimaging: A Systematic Literature Review. Comput. Methods Programs Biomed..

[13] Al-Shoukry S., Rassem T.H., Makbol N.M. (2020). Alzheimer’s Diseases Detection by Using Deep Learning Algorithms: A Mini-Review. IEEE Access.

[14] Petersen R.C., Aisen P.S., Beckett L.A., Donohue M.C., Gamst A.C., Harvey D.J., Jack C.R., Jagust W.J., Shaw L.M., Toga A.W. (2010). Alzheimer’s Disease Neuroimaging Initiative (ADNI): Clinical Characterization. Neurology.

[15] Marcus D.S., Wang T.H., Parker J., Csernansky J.G., Morris J.C., Buckner R.L. (2007). Open Access Series of Imaging Studies (OASIS): Cross-Sectional MRI Data in Young, Middle Aged, Nondemented, and Demented Older Adults. J. Cogn. Neurosci..

[16] Yang W., Lui R.L.M., Gao J.-H., Chan T.F., Yau S.-T., Sperling R.A., Huang X. (2011). Independent Component Analysis-Based Classification of Alzheimer’s Disease MRI Data. J. Alzheimer’s Dis..

[17] Bergh S., Holmen J., Gabin J., Stordal E., Fikseaunet A., Selbaek G., Saltvedt I., Langballe E.M., Tambs K. (2014). Cohort Profile: The Health and Memory Study (HMS): A Dementia Cohort Linked to the HUNT Study in Norway. Int. J. Epidemiol..

[18] Hojjati S.H., Ebrahimzadeh A., Khazaee A., Babajani-Feremi A. (2017). Predicting Conversion from MCI to AD Using Resting-State FMRI, Graph Theoretical Approach and SVM. J. Neurosci. Methods.

[19] Zhang Y., Wang S., Phillips P., Dong Z., Ji G., Yang J. (2015). Detection of Alzheimer’s Disease and Mild Cognitive Impairment Based on Structural Volumetric MR Images Using 3D-DWT and WTA-KSVM Trained by PSOTVAC. Biomed. Signal Process Control.

[20] Vichianin Y., Khummongkol A., Chiewvit P., Raksthaput A., Chaichanettee S., Aoonkaew N., Senanarong V. (2021). Accuracy of Support-Vector Machines for Diagnosis of Alzheimer’s Disease, Using Volume of Brain Obtained by Structural MRI at Siriraj Hospital. Front. Neurol..

[21] De Mendonça L.J.C., Ferrari R.J. (2023). Alzheimer’s Disease Classification Based on Graph Kernel SVMs Constructed with 3D Texture Features Extracted from MR Images. Expert. Syst. Appl..

[22] Sharma A., Kaur S., Memon N., Jainul Fathima A., Ray S., Bhatt M.W. (2021). Alzheimer’s Patients Detection Using Support Vector Machine (SVM) with Quantitative Analysis. Neurosci. Inform..

[23] Khan Y.F., Kaushik B., Chowdhary C.L., Srivastava G. (2022). Ensemble Model for Diagnostic Classification of Alzheimer’s Disease Based on Brain Anatomical Magnetic Resonance Imaging. Diagnostics.

[24] Khedher L., Illán I.A., Górriz J.M., Ramírez J., Brahim A., Meyer-Baese A. (2017). Independent Component Analysis-Support Vector Machine-Based Computer-Aided Diagnosis System for Alzheimer’s with Visual Support. Int. J. Neural Syst..

[25] Liu J., Wang J., Hu B., Wu F.-X., Pan Y. (2017). Alzheimer’s Disease Classification Based on Individual Hierarchical Networks Constructed with 3-D Texture Features. IEEE Trans. Nanobiosci..

[26] Long X., Chen L., Jiang C., Zhang L. (2017). Prediction and Classification of Alzheimer Disease Based on Quantification of MRI Deformation. PLoS ONE.

[27] Pasnoori N., Flores-Garcia T., Barkana B.D. (2024). Histogram-Based Features Track Alzheimer’s Progression in Brain MRI. Sci. Rep..

[28] Demirhan A. (2018). The Effect of Feature Selection on Multivariate Pattern Analysis of Structural Brain MR Images. Phys. Medica.

[29] Alshamlan H., Omar S., Aljurayyad R., Alabduljabbar R. (2023). Identifying Effective Feature Selection Methods for Alzheimer’s Disease Biomarker Gene Detection Using Machine Learning. Diagnostics.

[30] Cabral C., Morgado P.M., Campos Costa D., Silveira M. (2015). Predicting Conversion from MCI to AD with FDG-PET Brain Images at Different Prodromal Stages. Comput. Biol. Med..

[31] Schmitter D., Roche A., Maréchal B., Ribes D., Abdulkadir A., Bach-Cuadra M., Daducci A., Granziera C., Klöppel S., Maeder P. (2015). An Evaluation of Volume-Based Morphometry for Prediction of Mild Cognitive Impairment and Alzheimer’s Disease. Neuroimage Clin..

[32] Zhang Y., Wang S. (2015). Detection of Alzheimer’s Disease by Displacement Field and Machine Learning. PeerJ.

[33] Zhang Y., Dong Z., Phillips P., Wang S., Ji G., Yang J., Yuan T.-F. (2015). Detection of Subjects and Brain Regions Related to Alzheimer’s Disease Using 3D MRI Scans Based on Eigenbrain and Machine Learning. Front. Comput. Neurosci..

[34] Xu Y., Pan X., Zhou Z., Yang Z., Zhang Y. (2015). Structural Least Square Twin Support Vector Machine for Classification. Appl. Intell..

[35] Retico A., Bosco P., Cerello P., Fiorina E., Chincarini A., Fantacci M.E. (2015). Predictive Models Based on Support Vector Machines: Whole-Brain versus Regional Analysis of Structural MRI in the Alzheimer’s Disease. J. Neuroimaging.

[36] Ortiz A., Munilla J., Álvarez-Illán I., Górriz J.M., Ramírez J. (2015). Exploratory Graphical Models of Functional and Structural Connectivity Patterns for Alzheimer’s Disease Diagnosis. Front. Comput. Neurosci..

[37] Zhu Y., Zhu X., Kim M., Shen D., Wu G. Early Diagnosis of Alzheimer’s Disease by Joint Feature Selection and Classification on Temporally Structured Support Vector Machine. Proceedings of the Medical Image Computing and Computer-Assisted Intervention—MICCAI 2016.

[38] Khazaee A., Ebrahimzadeh A., Babajani-Feremi A. (2016). Application of Advanced Machine Learning Methods on Resting-State FMRI Network for Identification of Mild Cognitive Impairment and Alzheimer’s Disease. Brain Imaging Behav..

[39] Suk H.-I., Lee S.-W., Shen D. (2015). Latent Feature Representation with Stacked Auto-Encoder for AD/MCI Diagnosis. Brain Struct. Funct..

[40] Plocharski M., Østergaard L.R. (2016). Extraction of Sulcal Medial Surface and Classification of Alzheimer’s Disease Using Sulcal Features. Comput. Methods Programs Biomed..

[41] Tong T., Gray K., Gao Q., Chen L., Rueckert D. (2017). Multi-Modal Classification of Alzheimer’s Disease Using Nonlinear Graph Fusion. Pattern Recognit..

[42] Zhu X., Suk H.-I., Lee S.-W., Shen D. (2016). Subspace Regularized Sparse Multitask Learning for Multiclass Neurodegenerative Disease Identification. IEEE Trans. Biomed. Eng..

[43] Alam S., Kwon G. (2017). Alzheimer disease classification using KPCA, LDA, and multi-kernel learning SVM. Int. J. Imaging Syst. Technol..

[44] Beheshti I., Demirel H., Matsuda H. (2017). Classification of Alzheimer’s Disease and Prediction of Mild Cognitive Impairment-to-Alzheimer’s Conversion from Structural Magnetic Resource Imaging Using Feature Ranking and a Genetic Algorithm. Comput. Biol. Med..

[45] Tangaro S., Fanizzi A., Amoroso N., Bellotti R. (2017). A Fuzzy-Based System Reveals Alzheimer’s Disease Onset in Subjects with Mild Cognitive Impairment. Phys. Medica.

[46] Asgari M., Kaye J., Dodge H. (2017). Predicting Mild Cognitive Impairment from Spontaneous Spoken Utterances. Alzheimer’s Dement. Transl. Res. Clin. Interv..

[47] Beheshti I., Maikusa N., Daneshmand M., Matsuda H., Demirel H., Anbarjafari G. (2017). Classification of Alzheimer’s Disease and Prediction of Mild Cognitive Impairment Conversion Using Histogram-Based Analysis of Patient-Specific Anatomical Brain Connectivity Networks. J. Alzheimer’s Dis..

[48] Alam S., Kwon G.-R., Kim J.-I., Park C.-S. (2017). Twin SVM-Based Classification of Alzheimer’s Disease Using Complex Dual-Tree Wavelet Principal Coefficients and LDA. J. Healthc. Eng..

[49] Sun Z., Qiao Y., Lelieveldt B.P.F., Staring M. (2018). Integrating Spatial-Anatomical Regularization and Structure Sparsity into SVM: Improving Interpretation of Alzheimer’s Disease Classification. Neuroimage.

[50] Liu J., Li M., Lan W., Wu F.-X., Pan Y., Wang J. (2018). Classification of Alzheimer’s Disease Using Whole Brain Hierarchical Network. IEEE/ACM Trans. Comput. Biol. Bioinform..

[51] Zeng N., Qiu H., Wang Z., Liu W., Zhang H., Li Y. (2018). A New Switching-Delayed-PSO-Based Optimized SVM Algorithm for Diagnosis of Alzheimer’s Disease. Neurocomputing.

[52] Basaia S., Agosta F., Wagner L., Canu E., Magnani G., Santangelo R., Filippi M. (2019). Automated Classification of Alzheimer’s Disease and Mild Cognitive Impairment Using a Single MRI and Deep Neural Networks. Neuroimage Clin..

[53] Lahmiri S., Shmuel A. (2019). Performance of Machine Learning Methods Applied to Structural MRI and ADAS Cognitive Scores in Diagnosing Alzheimer’s Disease. Biomed. Signal Process Control.

[54] Kamathe R.S., Joshi K.R. (2018). A Novel Method Based on Independent Component Analysis for Brain MR Image Tissue Classification into CSF, WM and GM for Atrophy Detection in Alzheimer’s Disease. Biomed. Signal Process Control.

[55] Bi X., Shu Q., Sun Q., Xu Q. (2018). Random Support Vector Machine Cluster Analysis of Resting-State FMRI in Alzheimer’s Disease. PLoS ONE.

[56] Mazaheri A., Segaert K., Olichney J., Yang J.-C., Niu Y.-Q., Shapiro K., Bowman H. (2018). EEG Oscillations during Word Processing Predict MCI Conversion to Alzheimer’s Disease. Neuroimage Clin..

[57] Paraskevaidi M., Morais C.L.M., Halliwell D.E., Mann D.M.A., Allsop D., Martin-Hirsch P.L., Martin F.L. (2018). Raman Spectroscopy to Diagnose Alzheimer’s Disease and Dementia with Lewy Bodies in Blood. ACS Chem. Neurosci..

[58] Hett K., Ta V.-T., Manjón J.V., Coupé P. (2018). Adaptive Fusion of Texture-Based Grading for Alzheimer’s Disease Classification. Comput. Med. Imaging Graph..

[59] Peng J., Zhu X., Wang Y., An L., Shen D. (2019). Structured Sparsity Regularized Multiple Kernel Learning for Alzheimer’s Disease Diagnosis. Pattern Recognit..

[60] Fritsch J., Wankerl S., Noth E. Automatic Diagnosis of Alzheimer’s Disease Using Neural Network Language Models. Proceedings of the ICASSP 2019—2019 IEEE International Conference on Acoustics, Speech and Signal Processing (ICASSP).

[61] Gosztolya G., Vincze V., Tóth L., Pákáski M., Kálmán J., Hoffmann I. (2019). Identifying Mild Cognitive Impairment and Mild Alzheimer’s Disease Based on Spontaneous Speech Using ASR and Linguistic Features. Comput. Speech Lang..

[62] Bi X., Zhao X., Huang H., Chen D., Ma Y. (2020). Functional Brain Network Classification for Alzheimer’s Disease Detection with Deep Features and Extreme Learning Machine. Cogn. Comput..

[63] Irie R., Otsuka Y., Hagiwara A., Kamagata K., Kamiya K., Suzuki M., Wada A., Maekawa T., Fujita S., Kato S. (2020). A Novel Deep Learning Approach with a 3D Convolutional Ladder Network for Differential Diagnosis of Idiopathic Normal Pressure Hydrocephalus and Alzheimer’s Disease. Magn. Reson. Med. Sci..

[64] Dachena C., Casu S., Lodi M.B., Fanti A., Mazzarella G. Application of MRI, FMRI and Cognitive Data for Alzheimer’s Disease Detection. Proceedings of the 2020 14th European Conference on Antennas and Propagation (EuCAP).

[65] Burgos N., Colliot O. (2020). Machine Learning for Classification and Prediction of Brain Diseases: Recent Advances and Upcoming Challenges. Curr. Opin. Neurol..

[66] Sharma R., Goel T., Tanveer M., Murugan R. (2022). FDN-ADNet: Fuzzy LS-TWSVM Based Deep Learning Network for Prognosis of the Alzheimer’s Disease Using the Sagittal Plane of MRI Scans. Appl. Soft Comput..

[67] Arco J.E., Ortiz A., Castillo-Barnes D., Górriz J.M., Ramírez J. (2023). Ensembling Shallow Siamese Architectures to Assess Functional Asymmetry in Alzheimer’s Disease Progression. Appl. Soft Comput..

[68] Puri D.V., Nalbalwar S.L., Nandgaonkar A.B., Gawande J.P., Wagh A. (2023). Automatic Detection of Alzheimer’s Disease from EEG Signals Using Low-Complexity Orthogonal Wavelet Filter Banks. Biomed. Signal Process Control.

[69] Pirrone D., Weitschek E., Di Paolo P., De Salvo S., De Cola M.C. (2022). EEG Signal Processing and Supervised Machine Learning to Early Diagnose Alzheimer’s Disease. Appl. Sci..

[70] Cheng B., Liu M., Suk H.-I., Shen D., Zhang D. (2015). Multimodal Manifold-Regularized Transfer Learning for MCI Conversion Prediction. Brain Imaging Behav..

[71] Orouskhani M., Zhu C., Rostamian S., Shomal Zadeh F., Shafiei M., Orouskhani Y. (2022). Alzheimer’s Disease Detection from Structural MRI Using Conditional Deep Triplet Network. Neurosci. Inform..

[72] Chui K.T., Gupta B.B., Alhalabi W., Alzahrani F.S. (2022). An MRI Scans-Based Alzheimer’s Disease Detection via Convolutional Neural Network and Transfer Learning. Diagnostics.

[73] Sathish Kumar L., Hariharasitaraman S., Narayanasamy K., Thinakaran K., Mahalakshmi J., Pandimurugan V. (2022). AlexNet Approach for Early Stage Alzheimer’s Disease Detection from MRI Brain Images. Mater. Today Proc..

[74] Shanmugam J.V., Duraisamy B., Simon B.C., Bhaskaran P. (2022). Alzheimer’s Disease Classification Using Pre-Trained Deep Networks. Biomed. Signal Process Control.

[75] Wang S., Zhang Y., Dong Z., Du S., Ji G., Yan J., Yang J., Wang Q., Feng C., Phillips P. (2015). Feed-Forward Neural Network Optimized by Hybridization of PSO and ABC for Abnormal Brain Detection. Int. J. Imaging Syst. Technol..

[76] Gorji H.T., Haddadnia J. (2015). A Novel Method for Early Diagnosis of Alzheimer’s Disease Based on Pseudo Zernike Moment from Structural MRI. Neuroscience.

[77] Jha D., Kim J.-I., Kwon G.-R. (2017). Diagnosis of Alzheimer’s Disease Using Dual-Tree Complex Wavelet Transform, PCA, and Feed-Forward Neural Network. J. Healthc. Eng..

[78] Liu M., Zhang J., Adeli E., Shen D. (2019). Joint Classification and Regression via Deep Multi-Task Multi-Channel Learning for Alzheimer’s Disease Diagnosis. IEEE Trans. Biomed. Eng..

[79] Mahendran N., PM D.R.V. (2022). A Deep Learning Framework with an Embedded-Based Feature Selection Approach for the Early Detection of the Alzheimer’s Disease. Comput. Biol. Med..

[80] EL-Geneedy M., Moustafa H.E.-D., Khalifa F., Khater H., AbdElhalim E. (2023). An MRI-Based Deep Learning Approach for Accurate Detection of Alzheimer’s Disease. Alex. Eng. J..

[81] Lahmiri S. (2023). Integrating Convolutional Neural Networks, KNN, and Bayesian Optimization for Efficient Diagnosis of Alzheimer’s Disease in Magnetic Resonance Images. Biomed. Signal Process Control.

[82] Cicalese P.A., Li R., Ahmadi M.B., Wang C., Francis J.T., Selvaraj S., Schulz P.E., Zhang Y. (2020). An EEG-FNIRS Hybridization Technique in the Four-Class Classification of Alzheimer’s Disease. J. Neurosci. Methods.

[83] Ho T.K.K., Kim M., Jeon Y., Na E., Ullah Z., Kim B.C., Lee K.H., Song J., Kim J.G., Gwak J. (2021). Improving the Multi-class Classification of Alzheimer’s Disease with Machine Learning-based Techniques: An EEG-fNIRS Hybridization Study. Alzheimer’s Dement..

[84] Feng W., Van Halm-Lutterodt N., Tang H., Mecum A., Mesregah M.K., Ma Y., Li H., Zhang F., Wu Z., Yao E. (2020). Automated MRI-Based Deep Learning Model for Detection of Alzheimer’s Disease Process. Int. J. Neural Syst..

[85] Lopes M., Cassani R., Falk T.H. (2023). Using CNN Saliency Maps and EEG Modulation Spectra for Improved and More Interpretable Machine Learning-Based Alzheimer’s Disease Diagnosis. Comput. Intell. Neurosci..

[86] Odusami M., Maskeliūnas R., Damaševičius R. (2023). Pixel-Level Fusion Approach with Vision Transformer for Early Detection of Alzheimer’s Disease. Electronics.

[87] Yang F., Wang H., Wei S., Sun G., Chen Y., Tao L. (2023). Multi-Model Adaptive Fusion-Based Graph Network for Alzheimer’s Disease Prediction. Comput. Biol. Med..

[88] Rahim N., El-Sappagh S., Rizk H., El-serafy O.A., Abuhmed T. (2024). Information Fusion-Based Bayesian Optimized Heterogeneous Deep Ensemble Model Based on Longitudinal Neuroimaging Data. Appl. Soft Comput..

[89] Choudhury C., Goel T., Tanveer M. (2024). A Coupled-GAN Architecture to Fuse MRI and PET Image Features for Multi-Stage Classification of Alzheimer’s Disease. Inf. Fusion.

[90] Payan A., Montana G. (2015). Predicting Alzheimer’s Disease: A Neuroimaging Study with 3D Convolutional Neural Networks. arXiv.

[91] Ghazal M. (2018). Alzheimer’s Disease Diagnostics by a 3D Deeply Supervised Adaptable Convolutional Network. Front. Biosci..

[92] Suk H.-I., Wee C.-Y., Lee S.-W., Shen D. (2016). State-Space Model with Deep Learning for Functional Dynamics Estimation in Resting-State FMRI. Neuroimage.

[93] Ortiz A., Munilla J., Górriz J.M., Ramírez J. (2016). Ensembles of Deep Learning Architectures for the Early Diagnosis of the Alzheimer’s Disease. Int. J. Neural Syst..

[94] Aljovic A., Badnjevic A., Gurbeta L. Artificial Neural Networks in the Discrimination of Alzheimer’s Disease Using Biomarkers Data. Proceedings of the 2016 5th Mediterranean Conference on Embedded Computing (MECO).

[95] Zheng X., Shi J., Li Y., Liu X., Zhang Q. Multi-Modality Stacked Deep Polynomial Network Based Feature Learning for Alzheimer’s Disease Diagnosis. Proceedings of the 2016 IEEE 13th International Symposium on Biomedical Imaging (ISBI).

[96] Sarraf S., DeSouza D.D., Anderson J., Tofighi G. (2016). DeepAD: Alzheimer’s Disease Classification via Deep Convolutional Neural Networks Using MRI and FMRI. bioRxiv.

[97] Hon M., Khan N. Towards Alzheimer’s Disease Classification through Transfer Learning. Proceedings of the 2017 IEEE International Conference on Bioinformatics and Biomedicine (BIBM).

[98] Suk H.-I., Lee S.-W., Shen D. (2017). Deep Ensemble Learning of Sparse Regression Models for Brain Disease Diagnosis. Med. Image Anal..

[99] Ieracitano C., Mammone N., Bramanti A., Hussain A., Morabito F.C. (2019). A Convolutional Neural Network Approach for Classification of Dementia Stages Based on 2D-Spectral Representation of EEG Recordings. Neurocomputing.

[100] Lu D., Popuri K., Ding G.W., Balachandar R., Beg M.F. (2018). Multiscale Deep Neural Network Based Analysis of FDG-PET Images for the Early Diagnosis of Alzheimer’s Disease. Med. Image Anal..

[101] Li F., Liu M. (2018). Alzheimer’s Disease Diagnosis Based on Multiple Cluster Dense Convolutional Networks. Comput. Med. Imaging Graph..

[102] Cui R., Liu M., Li G. Longitudinal Analysis for Alzheimer’s Disease Diagnosis Using RNN. Proceedings of the 2018 IEEE 15th International Symposium on Biomedical Imaging (ISBI 2018).

[103] Spasov S., Passamonti L., Duggento A., Liò P., Toschi N. (2019). A Parameter-Efficient Deep Learning Approach to Predict Conversion from Mild Cognitive Impairment to Alzheimer’s Disease. Neuroimage.

[104] Wang H., Shen Y., Wang S., Xiao T., Deng L., Wang X., Zhao X. (2019). Ensemble of 3D Densely Connected Convolutional Network for Diagnosis of Mild Cognitive Impairment and Alzheimer’s Disease. Neurocomputing.

[105] Chitradevi D., Prabha S. (2020). Analysis of Brain Sub Regions Using Optimization Techniques and Deep Learning Method in Alzheimer Disease. Appl. Soft Comput..

[106] Dua M., Makhija D., Manasa P.Y.L., Mishra P. (2020). A CNN–RNN–LSTM Based Amalgamation for Alzheimer’s Disease Detection. J. Med. Biol. Eng..

[107] Lella E., Lombardi A., Amoroso N., Diacono D., Maggipinto T., Monaco A., Bellotti R., Tangaro S. (2020). Machine Learning and DWI Brain Communicability Networks for Alzheimer’s Disease Detection. Appl. Sci..

[108] Mehmood A., Maqsood M., Bashir M., Shuyuan Y. (2020). A Deep Siamese Convolution Neural Network for Multi-Class Classification of Alzheimer Disease. Brain Sci..

[109] Liu S., Yadav C., Fernandez-Granda C., Razavian N. On the Design of Convolutional Neural Networks for Automatic Detection of Alzheimer’s Disease. Proceedings of the Machine Learning for Health NeurIPS Workshop, USA.

[110] Xia Z., Yue G., Xu Y., Feng C., Yang M., Wang T., Lei B. A Novel End-to-End Hybrid Network for Alzheimer’s Disease Detection Using 3D CNN and 3D CLSTM. Proceedings of the 2020 IEEE 17th International Symposium on Biomedical Imaging (ISBI).

[111] Helaly H.A., Badawy M., Haikal A.Y. (2022). Toward Deep MRI Segmentation for Alzheimer’s Disease Detection. Neural Comput. Appl..

[112] Zhang C., Yang H., Fan C.-C., Chen S., Fan C., Hou Z.-G., Chen J., Peng L., Xiang K., Wu Y. (2023). Comparing Multi-Dimensional FNIRS Features Using Bayesian Optimization-Based Neural Networks for Mild Cognitive Impairment (MCI) Detection. IEEE Trans. Neural Syst. Rehabil. Eng..

[113] Jiao B., Li R., Zhou H., Qing K., Liu H., Pan H., Lei Y., Fu W., Wang X., Xiao X. (2023). Neural Biomarker Diagnosis and Prediction to Mild Cognitive Impairment and Alzheimer’s Disease Using EEG Technology. Alzheimers Res. Ther..

[114] Ruiz J., Mahmud M., Modasshir M., Shamim Kaiser M. (2020). 3D DenseNet Ensemble in 4-Way Classification of Alzheimer’s Disease. Brain Informatics.

[115] Pan D., Zeng A., Jia L., Huang Y., Frizzell T., Song X. (2020). Early Detection of Alzheimer’s Disease Using Magnetic Resonance Imaging: A Novel Approach Combining Convolutional Neural Networks and Ensemble Learning. Front. Neurosci..

[116] An N., Ding H., Yang J., Au R., Ang T.F.A. (2020). Deep Ensemble Learning for Alzheimer’s Disease Classification. J. Biomed. Inform..

[117] Fang X., Liu Z., Xu M. (2020). Ensemble of Deep Convolutional Neural Networks Based Multi-modality Images for Alzheimer’s Disease Diagnosis. IET Image Process.

[118] El-Sappagh S., Abuhmed T., Riazul Islam S.M., Kwak K.S. (2020). Multimodal Multitask Deep Learning Model for Alzheimer’s Disease Progression Detection Based on Time Series Data. Neurocomputing.

[119] Hedayati R., Khedmati M., Taghipour-Gorjikolaie M. (2021). Deep Feature Extraction Method Based on Ensemble of Convolutional Auto Encoders: Application to Alzheimer’s Disease Diagnosis. Biomed. Signal Process Control.

[120] Razzak I., Naz S., Ashraf A., Khalifa F., Bouadjenek M.R., Mumtaz S. (2022). Mutliresolutional Ensemble PartialNet for Alzheimer Detection Using Magnetic Resonance Imaging Data. Int. J. Intell. Syst..

[121] Abd El Hamid M.M., Shaheen M., Omar Y.M.K., Mabrouk M.S. (2023). Discovering Epistasis Interactions in Alzheimer’s Disease Using Integrated Framework of Ensemble Learning and Multifactor Dimensionality Reduction (MDR). Ain Shams Eng. J..

[122] Baglat P., Salehi A.W., Gupta A., Gupta G. (2020). Multiple Machine Learning Models for Detection of Alzheimer’s Disease Using OASIS Dataset. Re-Imagining Diffusion and Adoption of Information Technology and Systems: A Continuing Conversation. TDIT 2020. IFIP Advances in Information and Communication Technology.

[123] Khoei T.T., Catherine Labuhn M., Caleb T.D., Chen Hu W., Kaabouch N. A Stacking-Based Ensemble Learning Model with Genetic Algorithm for Detecting Early Stages of Alzheimer’s Disease. Proceedings of the 2021 IEEE International Conference on Electro Information Technology (EIT).

[124] Chatterjee S., Byun Y.-C. (2022). Voting Ensemble Approach for Enhancing Alzheimer’s Disease Classification. Sensors.

[125] Shaffi N., Hajamohideen F., Abdesselam A., Mahmud M., Subramanian K. (2022). Ensemble Classifiers for a 4-Way Classification of Alzheimer’s Disease. Applied Intelligence and Informatics.

